# Dehydroepiandrosterone in fibrotic interstitial lung disease: a translational study

**DOI:** 10.1186/s12931-022-02076-9

**Published:** 2022-06-08

**Authors:** Sabina A. Guler, Carlos Machahua, Thomas K. Geiser, Gregor Kocher, Thomas M. Marti, Benjamin Tan, Verdiana Trappetti, Christopher J. Ryerson, Manuela Funke-Chambour

**Affiliations:** 1 Department of Pulmonary Medicine, Inselspital, Bern University Hospital, University of Bern, Freiburgstrasse 18, 3010 Bern, Switzerland; 2 Department for BioMedical Research DBMR, Inselspital, Bern University Hospital, University of Bern, Bern, Switzerland; 3 Division of General Thoracic Surgery, Inselspital, Bern University Hospital, University of Bern, Bern, Switzerland; 4 Department of Medicine, University of British Columbia, Vancouver, Canada; 5 Institute of Anatomy, University of Bern, Bern, Switzerland; 6 Centre for Heart Lung Innovation, University of British Columbia, Vancouver, Canada

## Abstract

**Background:**

Dehydroepiandrosterone (DHEA) is a precursor sex hormone with antifibrotic properties. The aims of this study were to investigate antifibrotic mechanisms of DHEA, and to determine the relationship between DHEA-sulfate (DHEAS) plasma levels, disease severity and survival in patients with fibrotic interstitial lung diseases (ILDs).

**Methods:**

Human precision cut lung slices (PCLS) and normal human lung fibroblasts were treated with DHEA and/or transforming growth factor (TGF)-β1 before analysis of pro-fibrotic genes and signal proteins. Cell proliferation, cytotoxicity, cell cycle and glucose-6-phosphate dehydrogenase (G6PD) activity were assessed. DHEAS plasma levels were correlated with pulmonary function, the composite physiologic index (CPI), and time to death or lung transplantation in a derivation cohort of 31 men with idiopathic pulmonary fibrosis (IPF) and in an independent validation cohort of 238 men and women with fibrotic ILDs.

**Results:**

DHEA decreased the expression of pro-fibrotic markers in-vitro and ex-vivo. There was no cytotoxic effect for the applied concentrations, but DHEA interfered in proliferation by modulating the cell cycle through reduction of G6PD activity. In men with IPF (derivation cohort) DHEAS plasma levels in the lowest quartile were associated with poor lung function and higher CPI (adjusted OR 1.15 [95% CI 1.03–1.38], p = 0.04), which was confirmed in the fibrotic ILD validation cohort (adjusted OR 1.03 [95% CI 1.00–1.06], p = 0.01). In both cohorts the risk of early mortality was higher in patients with low DHEAS levels, after accounting for potential confounding by age in men with IPF (HR 3.84, 95% CI 1.25–11.7, p = 0.02), and for age, sex, IPF diagnosis and prednisone treatment in men and women with fibrotic ILDs (HR 3.17, 95% CI 1.35–7.44, p = 0.008).

**Conclusions:**

DHEA reduces lung fibrosis and cell proliferation by inducing cell cycle arrest and inhibition of G6PD activity. The association between low DHEAS levels and disease severity suggests a potential prognostic and therapeutic role of DHEAS in fibrotic ILD.

**Supplementary Information:**

The online version contains supplementary material available at 10.1186/s12931-022-02076-9.

## Introduction

Interstitial lung disease (ILD) is a group of inflammatory and fibrotic disorders that damage the lung parenchyma with various disease courses [1]. Especially patients with idiopathic pulmonary fibrosis (IPF) suffer from rapid disease progression and early mortality [2]. Other fibrotic ILDs including hypersensitivity pneumonitis (HP), connective tissue disease (CTD) associated ILD, and unclassifiable ILD can have similar progressive disease behaviour [3], and are characterized by reduced quality of life, frequent comorbidities, and age-related deficits [4]. Disease progression is difficult to predict in the individual patient but impacts treatment decisions.

Underlying mechanisms of progressive pulmonary fibrosis are still insufficiently understood. Biological and functional ageing are suggested to play a pathogenetic and perpetuating role in fibrotic ILDs [4, 5]. Patients with IPF are typically elderly men, an observation that has not been sufficiently explained yet [2]. Levels of dehydroepiandrosterone (DHEA) and its sulfated reservoir form (DHEAS) decrease with ageing, both have weak androgen effects and are precursors for androgens and estrogens, with additional biological roles (including immunosenescence) that have been proposed [6, 7].

Patients with IPF have lower plasma levels of DHEAS in comparison to healthy controls, and DHEA shows antifibrotic properties in-vitro [8]. A regulating role of DHEA in lung fibrosis is suggested by its anti-proliferative effect, regulation of extracellular matrix production [9], and effect on vascular remodelling [10]. In endothelial cells, DHEA inhibits cell growth by modulation of p53 and p21, proteins involved in the control of the cell cycle [6]; however, it is unknown if similar mechanisms apply to other cell types. Other hypothesized mechanisms by which DHEA interferes with cell growth and proliferation include apoptosis, autophagy [11, 12], and glucose-6-phosphate dehydrogenase (G6PD) inhibition [13, 14].

Despite recent advances in the management of patients with IPF and other ILDs with a progressive fibrotic phenotype [15, 16], pharmacological options remain limited and better approaches are urgently needed. In this translational study, we aimed to further investigate the previously described antifibrotic effect of DHEA in primary human lung fibroblasts in vitro and in an ex vivo model of human precision cut lung slices (PCLS) [8]. We hypothesized that DHEAS plasma levels would be associated with disease severity and survival in patients with fibrotic ILDs. We anticipated that these results would confirm the clinical importance of DHEAS for patients with fibrotic ILD and support further investigation into new potential therapeutic targets for this population.

## Methods

This translational project consisted of experimental and clinical components. Antifibrotic effects and mechanisms of DHEA were established in normal human lung fibroblasts and in complete human lung tissue (PCLS). Furthermore, the clinical role of DHEAS was explored in men with IPF, and in a second cohort of men and women with various fibrotic ILDs.

### In vitro and ex vivo experiments

#### DHEA treatment in vitro and ex vivo

Normal human lung fibroblasts (Lonza, Switzerland) (n = 3) were cultured in Ham’s F-12K (Kaighn’s) complete medium (Thermos Fisher Scientific, USA). At passage 4–6, cells were treated with 150 µM DHEA (Sigma-Aldrich, USA) and/or 5 ng/ml transforming growth factor (TGF)-β1 (R&D Systems, USA) in resting medium with 0.1% fetal bovine serum (RM). Experiments were performed independently, from different donors and in triplicates.

Gene (Additional file 1: Table S1) and protein expression of several pro-fibrotic markers and signalling proteins related to TGF-β1 signal pathway were evaluated by RT-qPCR and western blot. An ex vivo model for early fibrotic changes in the lung was established as described previously [17]. PCLS (400 µm) were obtained from healthy surrounding human lung tissue obtained during tumour resection and cut in a Compresstome® VF-310-0Z Vibrating Microtome (Precisionary, USA). PCLS were treated with 150 µM DHEA and/or a fibrotic cocktail during 48 h. Several pro-fibrotic markers were evaluated using RT-qPCR and immunofluorescence staining. Tissue sampling was approved by the local Ethics Committee, Bern, Switzerland (KEK-BE_2018-01801).

#### Proliferation and viability assay

CyQUANT™ XTT Cell Viability (Thermo Fisher Scientific) and LDH Cytotoxicity Assay (Thermo Fisher Scientific) were performed in human lung fibroblasts treated with different concentrations of DHEA (25, 50, 100, 150, 200 µM) at 24, 48 and 72 h, following the manufacturer’s protocol.

#### Cell cycle distribution and DNA damage

After treatment, lung fibroblasts were fixed with 4% paraformaldehyde and subsequently stained with diamidino-2-phenylindole (DAPI, Sigma-Aldrich) and a directly Alexa Fluor 488 conjugated mouse anti-γH2AX (Ser139) antibody (BioLegend, USA). Lung fibroblasts were analysed by flow cytometry BD LSRII SORP (Becton Dickinson, USA). Cell cycle stages and DNA damage was determined from the percentage of the total singlets (Additional file 1: Fig. S1), as previously described [18].

#### G6PD activity

G6PD activity assay (Sigma-Aldrich) was performed in normal human lung fibroblasts (n = 3) after 24 h of treatment with or without DHEA (150 µM) and TGF-β1, according to the manufacturer’s protocol. Additional details are provided in Additional file 1.

### Patient populations, measurements, and outcome assessment

The derivation cohort included men diagnosed with IPF from an ongoing prospective cohort study (Swiss Ethics Committee, Bern, approval number KEK 246/15 PB_2016-01524) [2].

The validation cohort included patients with a multidisciplinary diagnosis of fibrotic ILD from a Canadian outpatient ILD referral centre (UBC ethics board approval H10-03099). Fibrotic ILDs included IPF [2], fibrotic HP, unclassifiable ILD [1], and CTD-ILD [19]. All patients provided informed written consent.

#### DHEAS measurement in plasma

The samples from the derivation and validation cohort were analysed locally following the same procedure. Plasma samples were centrifuged and stored in aliquots in a biobank at − 80 °C, and enzyme-linked immunosorbent assay (ELISA) kits were used as per the manufacturer’s protocol (DRG International, Inc., Mountainside, NJ, USA). The assays were run in duplicate, and the mean value of the duplicates were used for analysis.

#### Outcome assessments

Pulmonary function tests were performed within 3 months of DHEAS measurement using established protocols [20, 21]. The Composite Physiologic Index (CPI) was calculated using forced vital capacity (FVC), forced expiratory volume in 1 s (FEV_1_), and diffusing capacity of the lung for carbon monoxide (DLCO). Originally, the CPI was developed to predict radiological severity of fibrosis and predicts mortality in ILD [22, 23]. Time to death, lung transplantation, or censoring was calculated from the date of blood draw for DHEAS measurement.

### Statistical analysis

Results from in vitro and ex vivo assays were reported as mean ± standard deviation (SD). Differences between experimental conditions were tested for statistical significance by one- or two-way analysis of variance (ANOVA) with post-hoc analyses, or the corresponding non-parametric tests when appropriate.

Patients were stratified by DHEAS plasma levels in the lowest quartile versus the second to fourth quartiles combined, this cut-off was chosen a-priori based on previous studies [24, 25]. Categorical variables were compared using chi-square or Fisher’s exact test, and continuous variables using two-sample t-test or Wilcoxon rank sum test depending on the distribution of the data. Pearson’s correlation was used for the unadjusted correlation between DHEAS, pulmonary function tests, and CPI. Odds ratios (OR) and corresponding 95% confidence intervals (95% CI) for the association of the lowest DHEAS quartile with pulmonary function tests and CPI were calculated using logistic regression models. Hazard ratios (HR) for the association between DHEAS and time to the composite outcome of death or lung transplantation were determined with Cox proportional hazard models. Models were adjusted for potential confounders with conceptual importance: age for the derivation cohort of men with IPF, and age, sex, IPF diagnosis, and prednisone treatment for the validation cohort (men and women with fibrotic ILDs). Proportional hazards assumption was tested using Schoenfeld residual tests. Unadjusted Kaplan Meier curves were used to illustrate the association of DHEAS with survival. There was no imputation performed for missing data. A two-sided p < 0.05 indicated statistical significance for all comparisons. Data were analysed using GraphPad Prism 9.0 (GraphPad Software Inc., USA) and R version 3.6.3 (R Foundation for Statistical Computing, Vienna, Austria).

## Results

### Antifibrotic mechanisms of DHEA

#### DHEA has strong antifibrotic effects in normal lung fibroblasts and in the fibrotic PCLS model

**Fig. 1:**
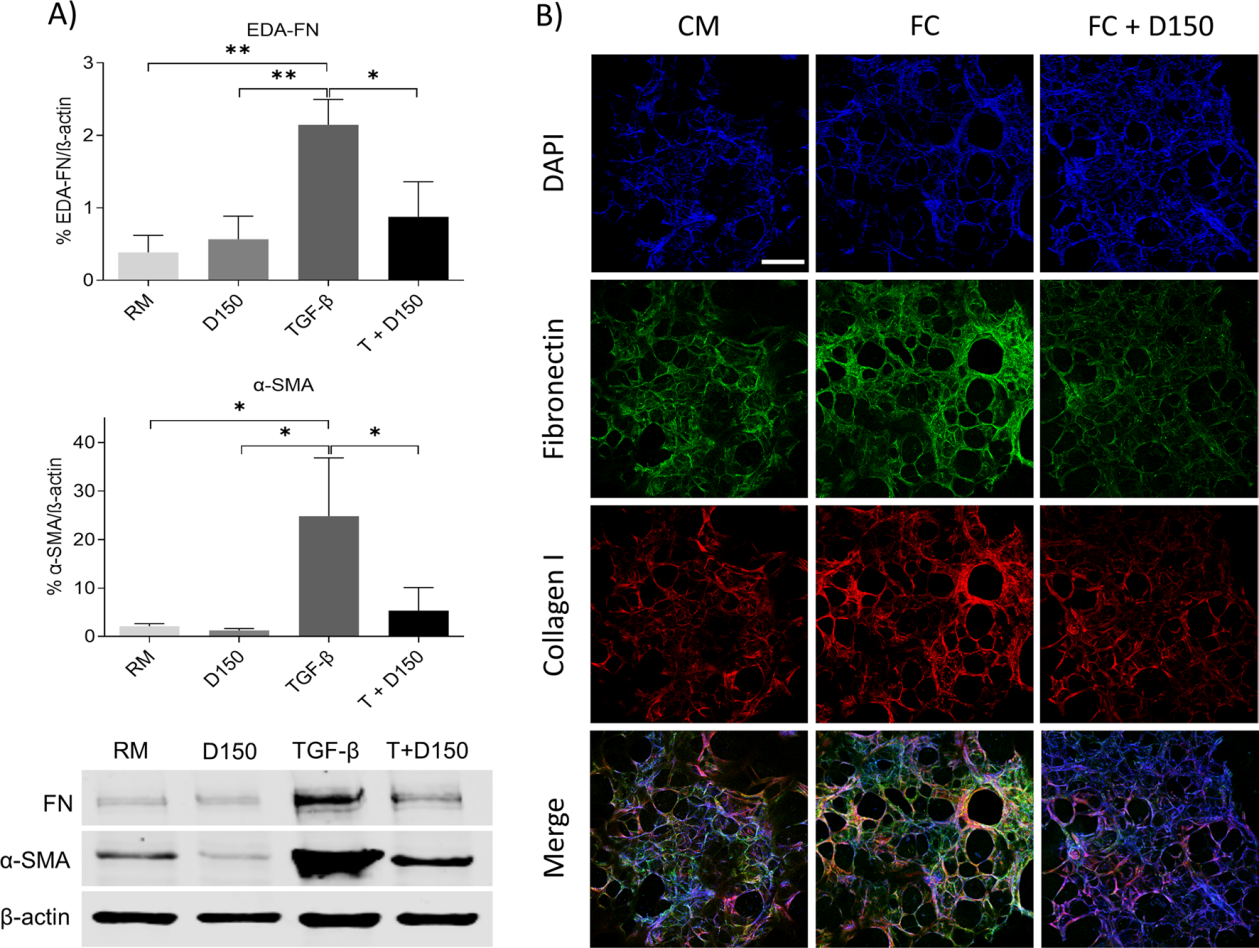
Antifibrotic effect of DHEA in vitro and ex vivo. **A** Protein expression of EDA-FN and α-SMA in normal human lung fibroblasts treated with DHEA 150 µM (D150) and/or TGF-β1 (5 ng/ml) (T + D150) for 48 h. Bars represent mean ± SD, (*) p < 0.05, (**) p < 0.01. **B** Immunofluorescence images of DAPI (blue), Fibronectin (green), collagen I (red), and merged in human PCLS treated with control medium (CM), fibrotic cocktail (FC) and FC plus DHEA (FC + D150). Scale bar = 500 µm. α-SMA: alpha smooth muscle actin; CM: control medium; DAPI: diamidino-2-phenylindole; D150, dehydroepiandrosterone 150 µM; EDA-FN: extra domain A fibronectin; FC: fibrotic cocktail; RM: resting medium; SD: standard deviation; TGF-β1: transforming growth factor β1; T + D150: TGF- β1 + DHEA 150 µM

In vitro, DHEA reduced extracellular matrix production such as EDA-fibronectin (EDA-FN) and fibroblast differentiation as shown by alpha smooth muscle actin (α-SMA) expression after 48 h (Fig. 1A). DHEA prevented the pro-fibrotic effect of TGF-β1 after 24 h of treatment, as observed in the reduction of the gene expression of profibrotic markers *EDA-FN*, *ACTA2*, *COL1A1* and *CTGF* (Additional file 1: Fig. S2).

Ex vivo, immunofluorescence images showed an increase of EDA-FN and Collagen I in PCLS treated with several pro-fibrotic mediators for 48 h. These early fibrotic changes could be prevented by DHEA (Fig. 1B and Additional file 1: Fig. S3). Furthermore, gene expression analyses of *EDA-FN*, *ACTA2*, and *COL1A1* showed that DHEA prevented the upregulation of fibrotic markers induced by pro-fibrotic treatment in this model (Additional file 1: Fig. S2).

#### The antifibrotic effect of DHEA is independent of Smad2/3 and Akt phosphorylation

**Fig. 2:**
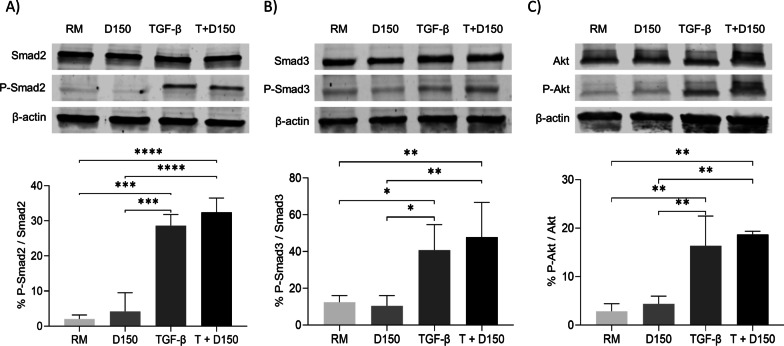
Effect of DHEA on Smad2/3 and Akt activation. Western blot analyses of P-Smad2/Smad2 (**A**), P-Smad3/Smad3 (**B**) and P-Akt/Akt ratio (**C**) in normal human lung fibroblasts treated for one hour with DHEA 150 µM (D150) and/or TGF-β1 (5 ng/ml) (T + D150). Bars show mean ± SD. (*) p < 0.05, (**) p < 0.01, (***) p < 0.001, (****) p < 0.0001. D150: dehydroepiandrosterone 150 µM; RM: resting medium; SD: standard deviation; TGF-β1: transforming growth factor β1, T + D150: TGF- β1 + DHEA 150 µM

To investigate specific interferences with TGF-β signalling pathway as a potential antifibrotic mechanism, Smad2/3, Akt and their phosphorylated forms were assessed by western blot. Figure 2 illustrates that DHEA did not interfere in Smad2/3 and Akt phosphorylation, suggesting that DHEA effects do not involve Smad or Akt phosphorylation downstream of TGF-β signalling.

#### DHEA interferes with cell proliferation by decreasing G6PD activity

**Fig. 3:**
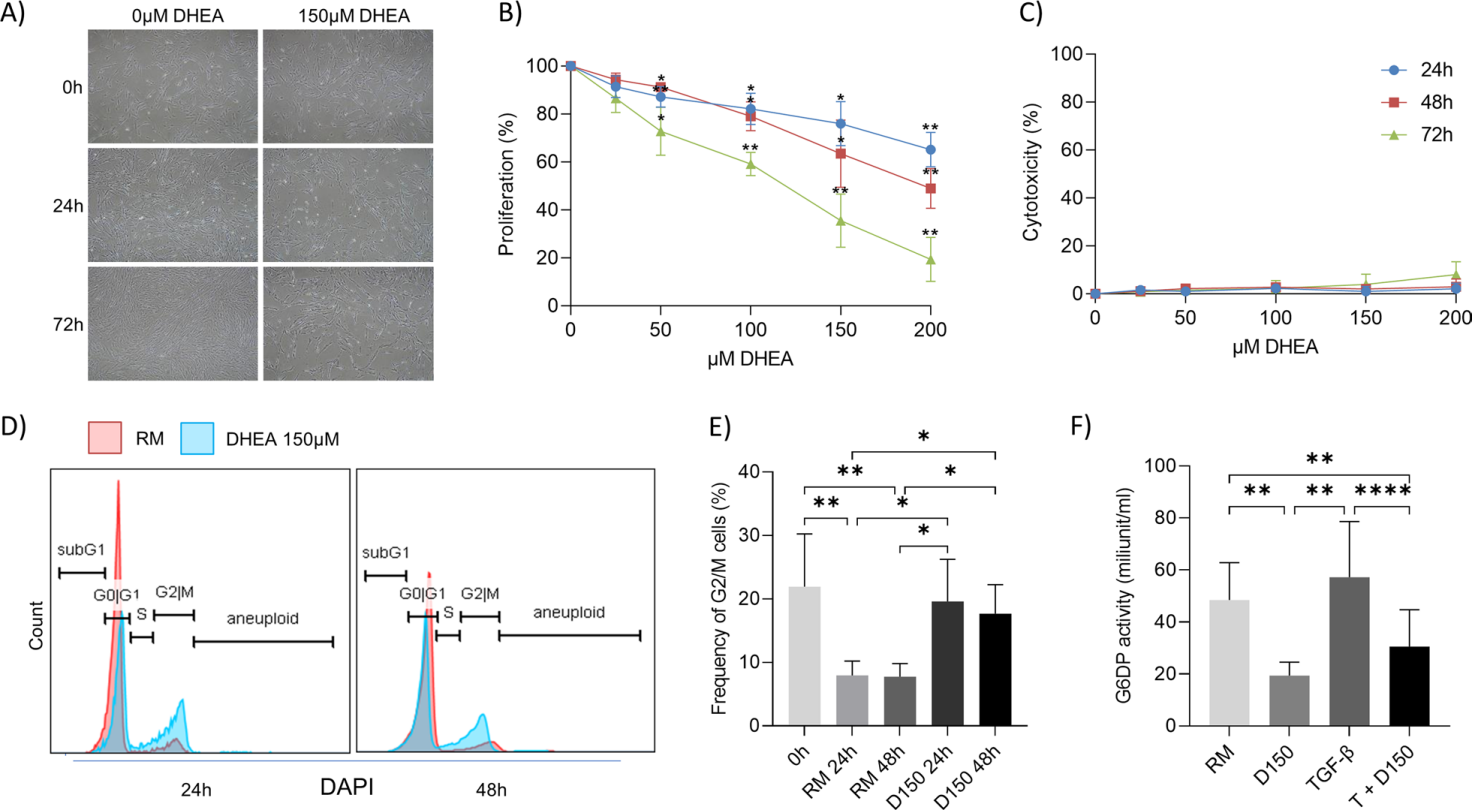
Effect of DHEA on cell growth and cell cycle in normal human lung fibroblasts. **A**, **B** DHEA has anti-proliferative effects in normal human lung fibroblasts in resting media (n = 3), **C** without cytotoxicity at concentrations of 25–200 µM compared to control at 24, 48 and 72 h. **D** Cell cycle distribution of normal human lung fibroblasts is shown at 24 h and 48 h after treatment with or without 150 µM DHEA (D150). **E** Frequency of lung fibroblasts in G2/M phase at the beginning of the experiment (0 h) and after 24–48 h of DHEA treatment. **F** G6PD activity is illustrated in normal human lung fibroblasts treated with 150 µM DHEA (D150), TGF-β or both (T + D150). Bars show mean + SD. (*) p < 0.05, (**) p < 0.01, (****) p < 0.0001. DHEA: dehydroepiandrosterone; D150, DHEA 150 µM; G6PD: glucose-6-phosphate dehydrogenase (G6PD); RM: resting medium; SD: standard deviation; TGF-β1/T: transforming growth factor β1; T + D150: TGF-β1 + DHEA 150 µM

DHEA reduced cell proliferation in a time- and dose-dependent manner, without increasing cell cytotoxicity (Fig. 3A). This anti-proliferative effect was linked to interference with the cell cycle regulation by DHEA. Flow cytometry analyses showed that DHEA promoted early cell cycle arrest in the G2/M phase population after 24 h of treatment compared to the control condition (Fig. 3B). The G2/M phase cell cycle arrest was not associated with DNA damage, as demonstrated by the unchanged frequency of γH2AX positive cells in all conditions (Additional file 1: Fig. S4B). To confirm the hypothesis, that DHEA mediates cell cycle arrest by altering the activity of G6PD and thus interfering in the nucleoside generation through pentose phosphate pathway, G6PD activity assay was performed in cell lysate from normal human lung fibroblasts treated with DHEA and TGF-β1. Lung fibroblasts had a reduced enzymatic activity of G6PD after 24 h of DHEA treatment (Fig. 3C).

### Patient characteristics

The derivation cohort included 31 men with IPF, with a mean (SD) age of 69.8 (10.3) and median (IQR) DHEAS plasma levels of 0.30 (0.19–0.58). Age, body mass index (BMI), smoking status, and antifibrotic treatment did not differ between patients with DHEAS levels in the lowest quartile compared to the remaining patients (Table 1).

**Table 1:** Patient characteristics in the derivation cohort (men with IPF) and the validation cohort (men and women with fibrotic ILD) stratified by DHEAS

	Derivation cohort (n = 31)		Validation cohort (n = 238)	
	DHEAS^a^ Lowest quartile	DHEAS^a^ 2nd–4th quartiles	DHEAS^b^ Lowest quartile	DHEAS^b^ 2nd–4th quartiles
Sex, men	8 (100%)	23 (100%)	17 (28%)	89 (50%)
Age	69.1 (13.8)	70.1 (9.1)	67.5 (10.6)	62.7 (13.2)
IPF diagnosis	8 (100%)	23 (100%)	5 (8%)	38 (21%)
BMI, kg/m^2^	29.0 (4.4)	28.0 (4.8)	28.8 (6.5)	26.9 (5.0)
Ever smoker	4 (50%)	15 (65%)	35 (58%)	112 (63%)
FVC, %-predicted	54.8 (16.1)	65.0 (15.5)	69.0 (21.6)	78.6 (16.8)
FEV1, %-predicted	65.5 (9.8)	74.7 (11.7)	70.1 (21.4)	80.7 (17.3)
DLCO, %-predicted	30.0 (9.8)	49.4 (15.5)	46.0 (18.2)	52.5 (15.4)
CPI	63.7 (11.9)	48.9 (12.5)	46.8 (15.5)	41.8 (12.8)
Nintedanib	3 (38%)	12 (52%)	1 (2%)	7 (4%)
Pirfenidone	3 (38%)	4 (17%)	1 (2%)	7 (4%)

Data shown are number (%), mean (SD), or median (IQR)

*BMI* body mass index, *CPI* composite physiologic index, *DHEAS* dehydroepiandrosterone sulfate, *DLCO* diffusing capacity of the lung for carbon monoxide, *FEV*_*1*_ forced expiratory volume in 1 s, *FVC* forced vital capacity, *ILD* interstitial lung disease, *IPF* idiopathic pulmonary fibrosis, *IQR* interquartile range, *SD* standard deviation

^a^Lowest quartile DHEAS < 0.19 µg/ml; 2nd–4th quartiles DHEAS ≥ 0.19 µg/ml

^b^Lowest quartile DHEAS < 0.20 µg/ml, 2nd–4th quartiles DHEAS ≥ 0.20 µg/ml

The validation cohort included 238 patients with fibrotic ILD. Forty-seven (20%) had unclassifiable ILD, 43 (18%) IPF, 24 (10%) fibrotic HP, 58 (24%) systemic sclerosis-associated ILD, 48 (20%) ILD associated with other CTDs, and 18 (8%) other ILDs. The 106 men and 132 women had median (IQR) DHEAS plasma levels of 0.50 (0.29–0.80) and 0.29 (0.14–0.58), respectively. Patients in the lowest DHEAS quartile were older, had less frequently IPF, and were more frequently treated with prednisone compared to the remaining patients. BMI, smoking status, and prednisone dose were not different between the two groups. Antifibrotic medications were prescribed in 7% of the entire cohort, with equal distribution across DHEAS quartiles (Table 1). Similarly, treatment with mycophenolate mofetil, azathioprine, rituximab, methotrexate, and other medications previously linked to DHEAS blood levels was not different between the groups (Additional file 1: Table S2).

### DHEAS and disease severity

**Fig. 4:**
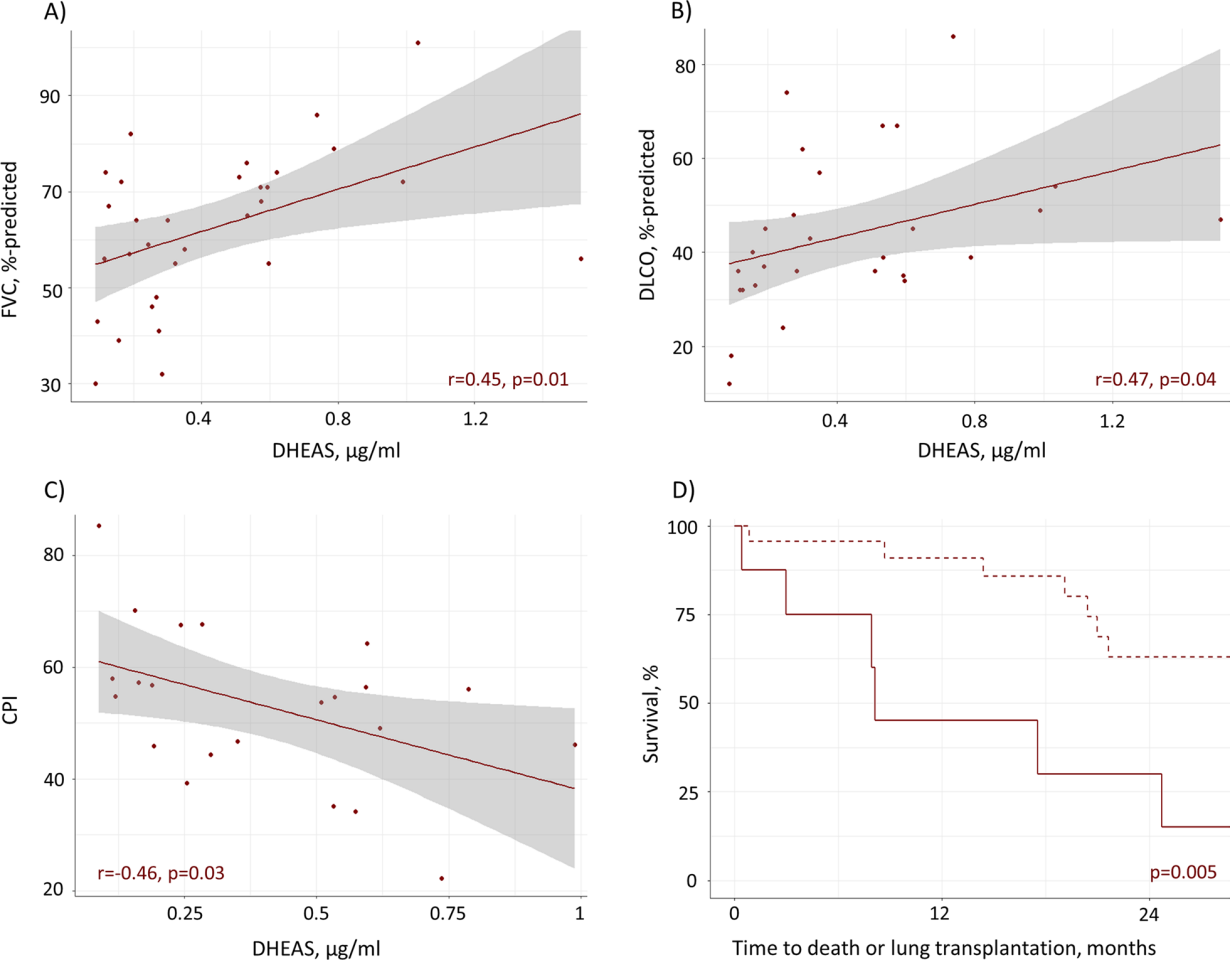
Relationship between DHEAS levels, pulmonary function, and survival in the derivation cohort of men with IPF. Correlation between DHEAS plasma levels (µg/ml) with FVC, %-predicted (**A**), DLCO, %-predicted (**B**), and CPI (**C**) in men with IPF. Kaplan Meier curves (**D**) for time to death or lung transplantation stratified by DHEAS < 0.19 µg/ml (solid line) versus DHEAS ≥ 0.19 µg/ml (dashed line), p-value from log rank test. CPI: composite physiologic index; DHEAS: dehydroepiandrosterone sulfate; DLCO: diffusing capacity of the lung for carbon monoxide; FVC: forced vital capacity; IPF: idiopathic pulmonary fibrosis

In the derivation cohort, patients with DHEAS levels in the lowest quartile had lower mean FVC (55 versus 65%-predicted, p = 0.14), lower DLCO (30 versus 49%-predicted, p = 0.0008), and higher CPI (64 versus 49, p = 0.03) compared to the remaining patients (Table 1). Correspondingly, there was a moderately strong correlation between DHEAS plasma levels and FVC %-predicted (r = 0.45, p = 0.01), DLCO %-predicted (r = 0.47, p = 0.04), and CPI (r = − 0.46, p = 0.03) (Fig. 4A–C). With adjustment for age, the relationship between low DHEAS and DLCO %-predicted remained statistically significant (OR 0.82 [95% CI 0.64–0.94], p = 0.03), with similar findings for CPI (OR 1.15 [95% CI 1.03–1.38], p = 0.04) (Table 2).

**Table 2:** Association of DHEAS in the lowest quartile with pulmonary function and survival in the derivation cohort (men with IPF)

DHEAS lowest quartile	Unadjusted		Adjusted for age	
	OR (95% CI)	p-value	OR (95% CI)	p-value
FVC, %	0.96 (0.90–1.01)	0.13	0.96 (0.90–1.01)	0.16
DLCO, %	0.84 (0.67–0.95)	0.03	0.82 (0.64–0.94)	0.03
CPI	1.12 (1.02–1.30)	0.05	1.15 (1.03–1.38)	0.04

	HR (95% CI)	p-value	HR (95% CI)	p-value
Survival^a^	4.35 (1.44–13.1)	0.009	3.84 (1.25–11.7)	0.02

E.g. every one % increase in FVC, %-predicted is associated with a 4% lower odds of a serum DHEAS value in the lowest quartile

*CI* confidence interval, *CPI* composite physiologic index, *DHEAS* dehydroepiandrosterone sulfate, *DLCO* diffusing capacity of the lung for carbon monoxide, *FVC* forced vital capacity, *HR* hazard ratio, *IPF* idiopathic pulmonary fibrosis, *OR* odds ratio

^a^Time to death or lung transplantation e.g. patients with DHEAS in the lowest quartile have a 4.35 higher hazard for early death or lung transplant compared to those with DHEAS values in the second to fourth quartile

**Fig. 5:**
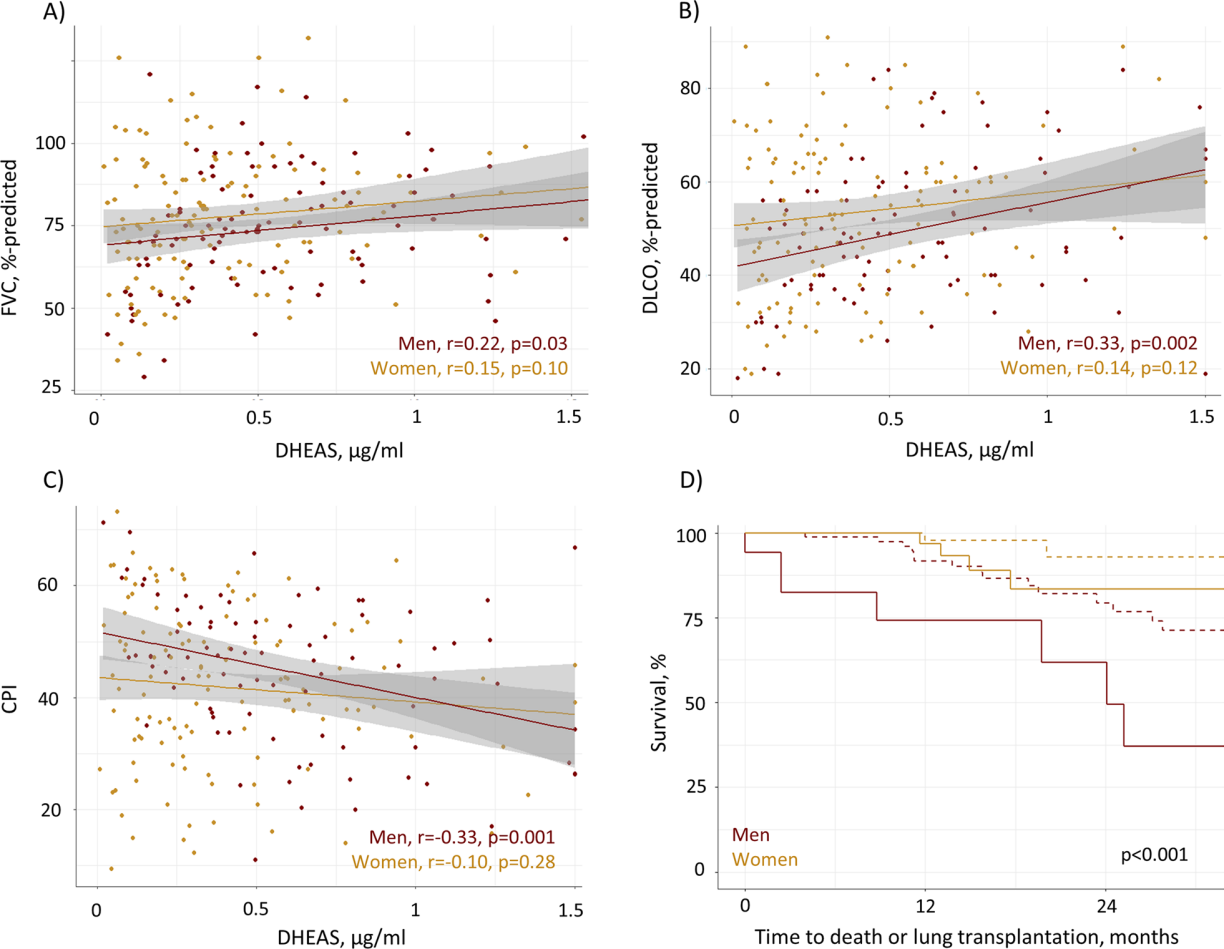
Relationship between DHEAS levels, pulmonary function, and survival in the validation cohort of men and women with fibrotic ILD. Correlation between DHEAS plasma levels (µg/ml) with FVC, %-predicted (**A**), DLCO, %-predicted (**B**), and CPI (**C**) in men and women with fibrotic ILD. Kaplan Meier curves (**D**) for time to death or lung transplantation stratified by sex and DHEAS < 0.20 µg/ml (solid line) versus DHEAS ≥ 0.20 µg/ml (dashed line), p-value from log rank test. CPI: composite physiologic index; DHEAS: dehydroepiandrosterone sulfate; DLCO: diffusing capacity of the lung for carbon monoxide; FVC: forced vital capacity; ILD: interstitial lung disease

In the validation cohort, patients with DHEAS levels in the lowest quartile had lower mean FVC (69 versus 79%-predicted, p = 0.002), lower DLCO (46 versus 53%-predicted, p = 0.01), and higher CPI (47 versus 42, p = 0.04) compared to the remaining patients (Table 1). DHEAS levels were correlated with pulmonary function in men with fibrotic ILD (FVC %-predicted r = 0.22, p = 0.03; DLCO %-predicted r = 0.33, p = 0.002; CPI r = − 0.34, p < 0.001), with non-significant correlations between DHEAS levels and pulmonary function in women (Fig. 5A–C). After adjustment for confounding by age, sex, IPF diagnosis, and prednisone treatment, the relationship between the lowest DHEAS quartile (< 0.20 µg/ml) and low DLCO and FVC %-predicted remained significant (both OR 0.97 [95% CI 0.95–0.99]; p = 0.004 and p = 0.02, respectively). The association of the lowest DHEAS quartile with high CPI was statistically significant, independent from age and sex (OR 1.03 [95% CI 1.01–1.06], p = 0.01), but not with additional adjustment for IPF and prednisone treatment (p = 0.08) (Table 3).

**Table 3:** Association of DHEAS in the lowest quartile with pulmonary function and survival in the validation cohort (men and women with fibrotic ILD)

DHEAS Lowest quartile	Unadjusted		Adjusted for			
			Age and sex		Age, sex, IPF and prednisone treatment	
	OR (95% CI)	p-value	OR (95% CI)	p-value	OR (95% CI)	p-value
FVC, %	0.97 (0.95–0.99)	< 0.001	0.96 (0.94–0.98)	< 0.001	0.97 (0.95–0.99)	0.004
DLCO, %	0.97 (0.95–0.99)	0.008	0.97 (0.95–0.99)	0.004	0.97 (0.95–0.99)	0.02
CPI	1.03 (1.00–1.05)	0.03	1.03 (1.01–1.06)	0.01	1.03 (1.00–1.06)	0.08

	HR (95% CI)	p-value	HR (95% CI)	p-value	HR (95% CI)	p-value
Survival^a^	1.96 (0.93–4.11)	0.07	2.30 (1.06–5.00)	0.03	3.17 (1.35–7.44)	0.008

E.g. every one % increase in FVC, %-predicted is associated with a 3% lower odds of a serum DHEAS value in the lowest quartile

*CI* confidence interval, *CPI* composite physiologic index, *DHEAS* dehydroepiandrosterone sulfate, *DLCO* diffusing capacity of the lung for carbon monoxide, *FVC* forced vital capacity, *HR* hazard ratio, *ILD* interstitial lung disease, *OR* odds ratio

^a^Time to death or lung transplantation e.g. patients with DHEAS in the lowest quartile have a 1.96 higher hazard for early death or lung transplant compared to those with normal/high DHEAS

### DHEAS and survival

Median (IQR) observation time in the derivation cohort was 20.4 (8.4–32.7) months, including 15 deaths and 1 lung transplant. Patients in the lowest DHEAS quartile had higher hazard of early death or lung transplant compared to the rest of the cohort (unadjusted HR 4.35, 95% CI 1.44–13.1, p = 0.009), independent of potential confounding by age (adjusted HR 3.84, 95% CI 1.25–11.7, p = 0.02) (Table 2, Fig. 4D).

In the validation cohort, median (IQR) observation time was 15.4 (7.9–21.7) months, including 30 deaths and 4 lung transplants. Patients in the lowest DHEAS quartile similarly had higher hazard of early death or lung transplantation compared to the rest of the cohort (HR adjusted for age and sex 2.3, 95% CI 1.06–5.0, p = 0.03). This relationship remained significant with additional adjustment for IPF diagnosis and prednisone treatment (HR 3.17, 95% CI 1.35–7.44, p = 0.008) (Table 3, Fig. 5D).

## Discussion

This study indicates a role of DHEA in lung fibrosis. Antifibrotic properties of DHEA were confirmed in vitro and shown for the first time ex vivo in a human model. Our findings suggest that DHEA inhibits cell proliferation and induces early cell cycle arrest by suppressing G6PD activity in lung fibroblasts. In men with IPF, higher DHEAS plasma levels correlate with preserved pulmonary function and lower risk for early mortality. These findings were replicated in a diverse validation cohort of men and women with fibrotic ILDs.

Previous studies have shown that DHEA decreases procollagen type I mRNA expression and protein synthesis in cultured cardiac fibroblasts, with prevention of oxidative stress and reduced cardiac fibrosis in animal studies [26, 27]. In lung fibroblasts, DHEA decreases proliferation in a dose- and time-dependent manner [8]. This was confirmed in the current study using normal human lung fibroblasts and, for the first time, in PCLS. We did not find an association of DHEA with Smad2/3 or Akt phosphorylation downstream of TGF-β, which suggests other antifibrotic downstream mechanisms. DHEA increases expression of several proteins involved in apoptosis, with some of these (e.g., p21) also involved in cell cycle regulation [6]. In our study, DHEA induced an early cell cycle arrest by inhibiting G6PD activity which might explain its anti-proliferative effects. Previously, inhibition of G6PD by DHEA has been mainly described in cancer cells [14, 28], in which high G6PD activity is one of the drivers of cell growth and proliferation [29]. In pulmonary fibrosis, controlling the excessive proliferation of fibroblasts and consequently reducing the profibrotic milieu is likely key in preventing progression.

DHEAS has been proposed as a protective factor against atherosclerosis, endothelial dysfunction, immunosenescence, and progression of liver fibrosis [10, 30]. DHEAS blood levels have been associated with overall and cardiovascular mortality in several population-based cohorts, with an overall stronger relationship between DHEAS deficiency and adverse outcomes in men than in women [7, 31]. In our cohort, the correlation between DHEAS levels, pulmonary fibrosis severity, and transplant-free survival was similarly stronger in men than in women. Consequently, the age associated decline in DHEAS might be one possible factor contributing to the increased risk for IPF and progressive pulmonary fibrosis in older men [32]. A previous study has measured levels of circulating DHEAS in pulmonary fibrosis (0.47 and 0.32 µg/ml in men and women with IPF, respectively), these were in line with our findings [8]. Decreased levels of DHEAS have also been observed in pulmonary hypertension [33, 34], and preclinical studies have demonstrated DHEAS to reverse right ventricular hypertrophy and vascular remodelling [10, 35]. Considering the common pathobiological mechanisms of pulmonary hypertension and pulmonary fibrosis, including dysregulated angiogenesis, endothelial dysfunction, and endothelial to mesenchymal transition [36, 37], inhibition of G6PD activity might be a common mechanism of impact of DHEA in pulmonary hypertension and fibrosis [38].

Our study has several strengths and limitations. This study exceeds previously demonstrated antifibrotic properties of DHEA [8] by proposing new underlying mechanisms, using an *ex vivo* human PCLS model which is considered a substitute for conventional models, and a better substitute model for human lung fibrosis than the classical mouse model with bleomycin. PCLS can be made from human lung and have preserved lung architecture with all pulmonary cell types available, which allows the investigation of changes in the extracellular matrix during fibrosis development [17]. However, this model was not designed to investigate severe fibrosis, reversal of fibrosis or effects of circulating blood cells. We did not exclude patients with subclinical pulmonary hypertension, and the inverse correlation between DHEAS and DLCO might be partly due to concurrent pulmonary vascular disease in patients with low DLCO. A definitive causality between DHEAS and progression of pulmonary fibrosis cannot be established from our clinical data. Nonetheless, the consistent association of DHEAS with FVC and CPI, the robustness of the findings to adjustment for potential confounders, and the antifibrotic effects of DHEAS suggest a pathogenetic role of DHEAS in fibrotic ILD. Lastly, the validation of our findings in two independent cohorts with different patient characteristics, and DHEAS plasma measurement in two different laboratories increase generalizability of the findings to different fibrotic ILD populations.

The safety of short-term DHEA supplementation has been established in several populations [39, 40]. For example, a small, non-controlled pilot study including patients with pulmonary hypertension associated with chronic obstructive pulmonary disease suggested efficacy of DHEA supplementation with a significant improvement in 6-min walk distance [39]. Based on the herein demonstrated antifibrotic effects of DHEA, and the relationship of DHEAS plasma levels with disease severity and adverse outcomes, future studies should investigate the effect of DHEA treatment on the progression of fibrosis in patients with ILD.

In summary, we establish a strong antifibrotic effect of DHEA in vitro and ex vivo with interference of DHEA in the fibroblast cell cycle by suppression of G6PD activity. Lower DHEAS plasma levels are associated with more severe disease and early mortality in men with IPF, and in an independent cohort of patients with fibrotic ILDs. These findings suggest DHEAS as a potential prognostic biomarker and therapeutic target in pulmonary fibrosis.

## Supplementary Information

**Additional file 1. Table S1**. List of qPCR primers.** Table S2**. Medication stratified by DHEAS in the lowest quartile compared to the second to fourth quartile combined in the validation cohort.** Figure S1**. Flow cytometry framework. Lung fibroblasts were separated from cell debris and analyses was made based on single cells. Cell cycle distribution was evaluated following DAPI staining. DNA damage was estimated from single cells by the presence of γH2AX (negative control = control medium (CM); positive control = 1mM H_2_O_2_).** Figure S2**. Gene expression of fibrotic markers* in vitro* and* ex vivo* after DHEA. A) Fibrotic markers* EDA-FN*,* ACTA2*,* COL1A1* and* CTGF* from normal human lung fibroblasts incubated in vitro with TGF-β1 and/or DHEA (D150) (T + D150). B)* Ex vivo* gene expression of PCLS stimulated with a fibrotic cocktail (FC), DHEA (D150) or both (FC + D150). Bars show mean ± SD. (*) p<0.05, (**) p<0.01, (***) p<0.001, (****) p<0.0001.** Figure S3**. Immunofluorescence staining of EDA-fibronectin and collagen I in PCLS treated with/without DHEA and the fibrotic cocktail. The signal of EDA-fibronectin (EDA-FN, green) and collagen I (red) in PCLS treated with fibrotic cocktail was reduced after addition of DHEA (FC + D150). Nucleus staining with DAPI (blue). Pictures were taken at 10X magnification. (–) scale represent 500 µm.** Figure S4**. Effect of DHEA on the cell cycle and DNA damage. A) Cell cycle distribution of control lung fibroblasts (n=3) at the beginning of the experiment (0h) and after treatment with DHEA (D150) or in resting medium (RM) for 24 and 48h. B) Frequency of γH2AX+ cells in lung fibroblasts treated with/without DHEA. (****) p<0.0001. compared to the positive control of DNA damage (H_2_O_2_ 1mM).

## Data Availability

The datasets used for the current study are available from the corresponding author on reasonable request.

## Abbreviations

α-SMA: Alpha smooth muscle actin
ANOVA: Analysis of variance
BMI: Body mass index
CI: Confidence interval
CPI: Composite Physiologic Index
CTD: Connective tissue disease
DAPI: Diamidino-2-phenylindole
DHEA: Dehydroepiandrosterone
DHEAS: Dehydroepiandrosterone sulfate
DLCO: Diffusing capacity of the lung for carbon monoxide
EDA-FN: EDA-fibronectin
ELISA: Enzyme-linked immunosorbent assay
FEV_1_: Forced expiratory volume in 1 s
FVC: Forced vital capacity
G6PD: Glucose-6-phosphate dehydrogenase
HP: Hypersensitivity pneumonitis
HR: Hazard ratio
ILD: Interstitial lung disease
IPF: Idiopathic pulmonary fibrosis
IQR: Interquartile range
OR: Odds ratios
PCLS: Precision cut lung slices
SD: Standard deviation
TGF: Transforming growth factor
