# Supplementary material for: Dehydroepiandrosterone in fibrotic interstitial lung disease: a translational study
Source: Respir Res. 2022 Jun 8;23:149. doi: 10.1186/s12931-022-02076-9 (PMC9178848; doi:10.1186/s12931-022-02076-9)
Supplement: Supplementary file 1 — Additional file 1. Table S1. List of qPCR primers. Table S2. Medication stratified by DHEAS in the lowest quartile compared to the second to fourth quartile combined in the validation cohort. Figure S1. Flow cytometry framework. Lung fibroblasts were separated from cell debris and analyses was made based on single cells. Cell cycle distribution was evaluated following DAPI staining. DNA damage was estimated from single cells by the presence of γH2AX (negative control = control medium (CM); positive control = 1mM H2O2). Figure S2. Gene expression of fibrotic markers in vitro and ex vivo after DHEA. A) Fibrotic markers EDA-FN, ACTA2, COL1A1 and CTGF from normal human lung fibroblasts incubated in vitro with TGF-β1 and/or DHEA (D150) (T + D150). B) Ex vivo gene expression of PCLS stimulated with a fibrotic cocktail (FC), DHEA (D150) or both (FC + D150). Bars show mean ± SD. (*) p<0.05, (**) p<0.01, (***) p<0.001, (****) p<0.0001. Figure S3. Immunofluorescence staining of EDA-fibronectin and collagen I in PCLS treated with/without DHEA and the fibrotic cocktail. The signal of EDA-fibronectin (EDA-FN, green) and collagen I (red) in PCLS treated with fibrotic cocktail was reduced after addition of DHEA (FC + D150). Nucleus staining with DAPI (blue). Pictures were taken at 10X magnification. (–) scale represent 500 µm. Figure S4. Effect of DHEA on the cell cycle and DNA damage. A) Cell cycle distribution of control lung fibroblasts (n=3) at the beginning of the experiment (0h) and after treatment with DHEA (D150) or in resting medium (RM) for 24 and 48h. B) Frequency of γH2AX+ cells in lung fibroblasts treated with/without DHEA. (****) p<0.0001. compared to the positive control of DNA damage (H2O2 1mM). [file 12931_2022_2076_MOESM1_ESM.docx]

# **ADDITIONAL FILE 1**

# **Dehydroepiandrosterone in fibrotic interstitial lung disease – a translational study**

Sabina A. Guler^1,2^, Carlos Machahua^1,2^, Thomas K. Geiser^1,2^, Gregor Kocher^2,3^, Thomas M. Marti^2,3^, Benjamin Tan^4^, Verdiana Trappetti^5^, Christopher J. Ryerson^4,6^, Manuela Funke-Chambour^1,2^

^1^Department of Pulmonary Medicine, Inselspital, Bern University Hospital, University of Bern, Switzerland.

^2^Department for BioMedical Research DBMR, Inselspital, Bern University Hospital, University of Bern, Switzerland.

^3^Division of General Thoracic Surgery, Inselspital, Bern University Hospital, University of Bern, Switzerland.

^4^Department of Medicine, University of British Columbia, Vancouver, Canada.

^5^Institute of Anatomy, University of Bern, Bern, Switzerland.

^6^Centre for Heart Lung Innovation, University of British Columbia, Vancouver, Canada.

**METHODS**

**In vitro and ex vivo experiments**

*DHEA treatment in vitro and ex vivo*

Anti-fibrotic effects of dehydroepiandrosterone (DHEA) (150µM, Sigma-Aldrich) were tested in an *in vitro* model of normal human lung fibroblasts (n=3, passage 4-6) (Lonza, Switzerland) stimulated with 5ng/ml transforming growth factor beta 1 (TGF-β1, R&D Systems) in Ham's F-12K (Kaighn's) medium (Thermo Fisher Scientific) resting media (0% fetal bovine serum [FBS] Gibco, Thermo Fisher Scientific). In addition, an *ex vivo* model of human fibrotic precision cut lung slices (PCLS) was used. Control lung tissue (n=6) was obtained from distal non-tumorous areas of lung resections from patients who underwent surgery for tumour resection (approved by the local Ethical Committee, ref. KEK-BE_2018-01801). Lung tissue was infiltrated with 2% low gelling temperature agarose solution (Sigma–Aldrich) in culture medium. After agarose gelation, small cubes of lung tissue were embedded in 4% agarose solution and cut in thin slices (400 µm) in a Compresstome® VF-310-0Z Vibrating Microtome (Precisionary). PCLS were cultured in high glucose Dulbecco's Modified Eagle Medium (DMEM; Gibco; Thermo Fisher Scientific) supplemented with antibiotic-antimycotic solution 1X (Gibco, Thermo Fisher Scientific), 0.2M 4-(2-hydroxyethyl)-1-piperazineethanesulfonic acid (HEPES) buffer (Gibco; Thermo Fisher Scientific) and 10% FBS.

PCLS were treated with 150µM DHEA in DMEM 0.1% FBS with or without a fibrotic cocktail (5ng/ml TGF-β1, 5µM lysophosphatidic acid, 10ng/ml platelet-derived growth factor-AB, and 10ng/ml tumour necrosis factor-α).

*Western blot analysis*

After incubation with/without DHEA and TGF-β1, cells were collected and lysated with radioimmunoprecipitation assay (RIPA) buffer (Thermo Fisher Scientific) plus proteinase (Sigma–Aldrich) and phosphatase inhibitor (Thermo Fisher Scientific). Pierce™ bicinchoninic acid (BCA) Protein Assay Kit (Thermo Fisher Scientific) was used for measuring protein content. Samples were run in Any kD™ Mini-PROTEAN® TGX™ Precast Protein Gels (Bio-Rad) and transferred in a Trans-Blot Turbo Mini 0.2 µm Nitrocellulose Transfer Packs (Bio-Rad). Membranes were blocked and incubated with the antibodies Mouse monoclonal anti EDA-FN (ab6328, Abcam), Mouse monoclonal anti αSMA (A2547, Sigma-Aldrich), Rabbit monoclonal anti Smad2 (3122, Cell Signalling Technology), Rabbit polyclonal anti p-Smad2 (Ser465/467) (3101, Cell Signalling Technology), Rabbit monoclonal anti Smad3 (9523, Cell Signalling Technology), Rabbit monoclonal anti p-Smad3 (Ser423/425) (9520, Cell Signalling Technology), Rabbit polyclonal anti Akt (9272, Cell Signalling Technology), Rabbit monoclonal anti p-Akt (Ser473) (4060, Cell Signalling Technology), Mouse monoclonal anti β-Actin (926-42212, LI-COR, USA), Rabbit monoclonal anti β-Actin (926-42210, LI-COR). As secondary antibodies, we used IRDye 800CW Donkey anti-Mouse IgG (H + L) (926-32212, LI-COR) and IRDye 680RD Goat anti-Rabbit IgG (H+L) (926-68071, LI-COR). Images were obtained in a LI-COR Odyssey imager (LI-COR) by fluorescence and semi-quantitative analysis was made with image Studio lite Vr5.2 (Li-COR).

*Immunofluorescence staining*

After treatment, PCLS were washed with phosphate-buffered saline (PBS) and fixed in 4% paraformaldehyde (PFA) for immunofluorescence staining. Permeabilization and blocking were performed before incubating O/N with primary antibodies against fibronectin (Mouse monoclonal anti EDA-FN, Abcam) and collagen I (Rabbit monoclonal anti COL1A1 (E6A8E), 39952S, Cell Signalling Technology). The next day, PCLS were washed and incubated for 3 hours with secondary goat antibodies against mouse IgG (A-11029, Thermo Fisher Scientific) and rabbit IgG (A-11010, Thermo Fisher Scientific) antibodies as well as diamidino-2-phenylindole (DAPI) for nuclear staining (Sigma-Aldrich). PCLS were mounted in glass bottom dishes (MatTek Life Sciences) and imaged with a Zeiss LSM 710 confocal microscope (Carl Zeiss AG). Images are represented as Maximum Intensity Projection of 3x3-tile regions z-stacks (140µm of thickness).

*Gene expression*

RNA samples were isolated from normal human lung fibroblasts after 24h of treatment with NucleoSpin® RNA isolation kit (Macherey-Nagel, Germany), following the manufacturer’s instructions. Reverse transcription of mRNA was performed with Omniscript RT Kit (Qiagen) and cDNA samples were blended with Fast SYBR™ Green Master Mix (Thermo Fisher Scientific). Gene expression of *EDA-FN*, *ACTA2*, *COL1A1*, *CTGF*, with *B2M* as housekeeping gene (sequence in **Table S1**) were evaluated by real-time qPCR using Applied Biosystems™ 7500 Fast Real-time PCR System (Thermo Fisher Scientific).

RNA samples from PCLS were obtained by two consecutive procedures: TRIzol™ Reagent (Thermo Fisher Scientific) and RNA Clean & Concentrator™-5 (Zymo Research), according to the producer’s protocol. RT-PCR and real time qPCR for *EDA-FN*, *ACTA2*, *COL1A1*, and *B2M* as housekeeping were performed following the method described previously.

*Proliferation and cytotoxicity assay*

To determine cell proliferation, primary lung fibroblasts (n=3) were seeded in a 96-well plate (2,000 cells/well) and 100µl of complete medium. Cells were treated with five different concentrations of DHEA (25, 50, 100, 150 and 200µM). CyQUANT™ XTT Cell Viability Assay (Thermo Fisher Scientific) was performed at 24, 48 and 72h after treatment.

To analyse DHEA cytotoxicity, cells were treated at the same time-dose experiments described above and CyQUANT™ LDH Cytotoxicity Assay (Thermo Fisher Scientific) was performed. Experiments were performed in triplicates. Plates were read in a microplate reader Infinite® M1000 (Tecan) at the corresponding wavelength. Results obtained from DHEA treatment at each experimental time point were shown in percentage compared with control conditions (0µM DHEA) assuming that this represented a 100% of proliferation and 0% of cytotoxicity.

*Cell cycle distribution and DNA damage*

One million lung fibroblast were seeded in a 10cm diameter petri dish. After reaching 70-80% of confluence, cells were treated with/without 150µM DHEA in resting condition. Cells were harvested after 24 and 48h of incubation. To observe cell cycle distribution, lung fibroblasts were stained with DAPI and passed through a BD LSR II Flow Cytometer (Becton Dickinson). Cell cycle stage was determinate from the percentage of the total singlets following the diagram in **Figure S1**.

To evaluate possible DNA damage, cells were stained with mouse anti-γH2AX (Ser139) conjugated with Alexa Fluor 488 (BioLegend) and the frequency of positive cells were quantified by flow cytometry measurement. For positive control of DNA damage, cells were incubated in 1mM H_2_O_2_ during 1h.

*G6PD activity*

G6PD activity assay (Sigma-Aldrich) was performed in normal human lung fibroblasts (n=3) after 24h of treatment with or without DHEA (150µM) and TGF-β1, following the manufacturer’s recommendations. Briefly, 1 million cells were lysated in the corresponding assay buffer and 50µg protein of each sample was incubated with the master reaction mix in a 96-well plate. Kinetic reaction was performed in duplicates until any sample reached the maximum absorbance of the standard curve at 450nm. Results of the enzymatic activity were expressed in milliunit/ml.

**TABLES**

**Table S1**. **List of qPCR primers.**

| Gene | Sequence |
| --- | --- |
| *B2M* forward | CTCCGTGGCCTTAGCTGTG |
| *B2M* reverse | TTTGGAGTACGCTGGATAGCCT |
| *EDA-FN* forward | TAAAGGACTGGCATTCACTGA |
| *EDA-FN* reverse | GTGCAAGGCAACCACACTGAC |
| *ACTA2* forward | CAGGGCTGTTTTCCCATCCAT |
| *ACTA2* reverse | GCCATGTTCTATCGGGTACTTC |
| *COL1A1* forward | CCAGAAGAACTGGTACATCAGCA |
| *COL1A1* reverse | CGCCATACTCGAACTGGGAAT |
| *CTGF* forward | GGCAAAAAGTGCATCCGTACT |
| *CTGF* reverse | CCGTCGGTACATACTCCACAG |

**Table S2. Medication stratified by DHEAS in the lowest quartile compared to the second to fourth quartile combined in the validation cohort.**

| Medications | All  (n=238) | DHEAS  lowest quartile (n=60) | DHEAS  2^nd^ - 4^th^ quartiles (n=178) |
| --- | --- | --- | --- |
| Nintedanib | 8 (3%) | 1 (2%) | 7 (4%) |
| Pirfenidone | 8 (3%) | 1 (2%) | 7 (4%) |
| Prednisone | 53 (22%) | 31 (52%) | 22 (12%) |
| Prednisone, dose* | 8 (5-10) | 7.5 (5-10) | 10 (5-16.7) |
| Mycophenolate mofetil | 68 (29%) | 22 (37%) | 46 (26%) |
| Azathioprine | 15 (6%) | 4 (7%) | 11 (6%) |
| Rituximab | 7 (3%) | 4 (7%) | 3 (2%) |
| Methotrexate | 6 (3%) | 1 (2%) | 5 (3%) |
| Benzodiazepine | 8 (3%) | 3 (5%) | 5 (3%) |
| Statin | 56 (24%) | 16 (27%) | 40 (22%) |
| Metformin | 17 (7%) | 3 (5%) | 14 (8%) |
| Insulin | 4 (2%) | 1 (2%) | 3 (2%) |
| Calcium-Antagonist | 46 (19%) | 10 (17%) | 36 (20%) |
| Estrogen | 7 (3%) | 3 (5%) | 4 (2%) |

DHEAS levels <0.20 µg/ml are in the lowest quartile.

*in patients with prednisone treatment

**FIGURES**

**Figure S1.** **Flow cytometry framework.** Lung fibroblasts were separated from cell debris and analyses was made based on single cells. Cell cycle distribution was evaluated following DAPI staining. DNA damage was estimated from single cells by the presence of γH2AX (negative control = control medium (CM); positive control = 1mM H_2_O_2_).

**
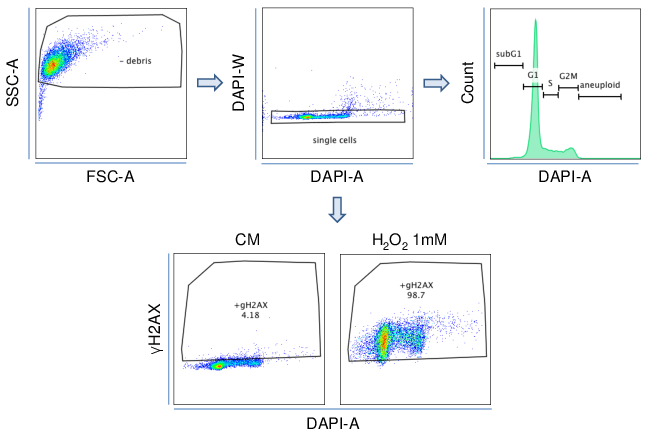
**

**Figure S2. Gene expression of fibrotic markers *in vitro* and *ex vivo* after DHEA.** A) Fibrotic markers *EDA-FN*, *ACTA2*, *COL1A1* and *CTGF* from normal human lung fibroblasts incubated *in vitro* with TGF-β1 and/or DHEA (D150) (T + D150). B) *Ex vivo* gene expression of PCLS stimulated with a fibrotic cocktail (FC), DHEA (D150) or both (FC + D150).

Bars show mean ± SD. (*) p<0.05, (**) p<0.01, (***) p<0.001, (****) p<0.0001.


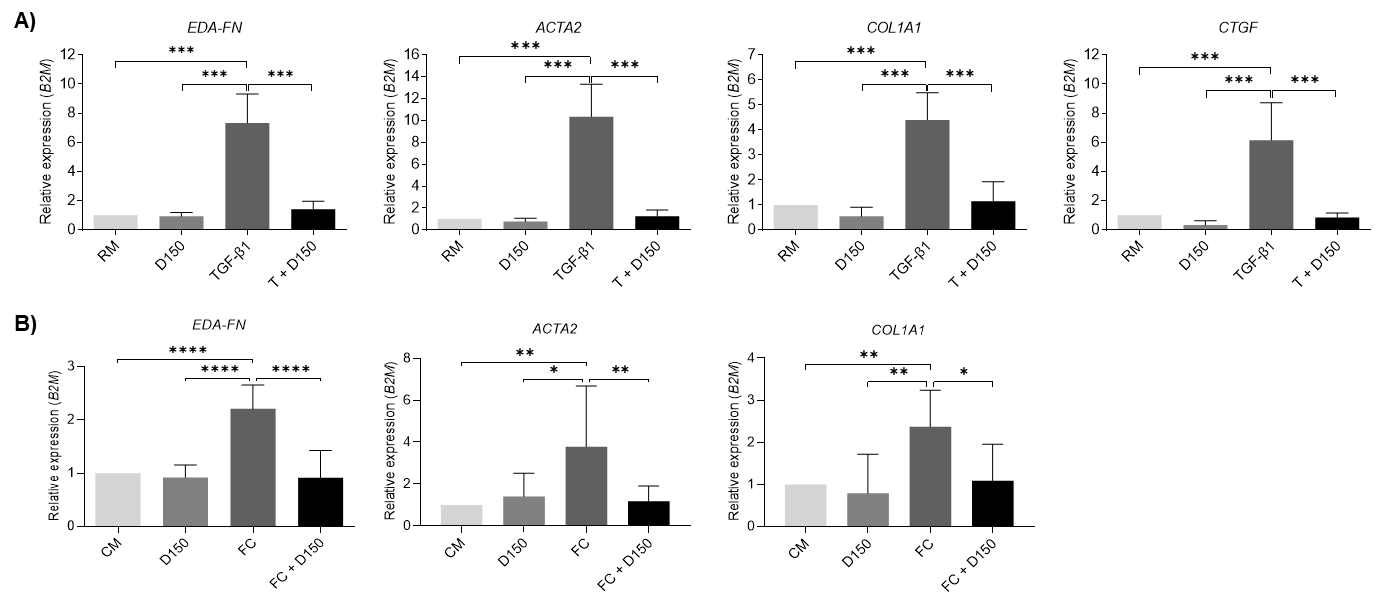


**Figure S3.** **Immunofluorescence staining of EDA-fibronectin and collagen I in PCLS treated with/without DHEA and the fibrotic cocktail.** The signal of EDA-fibronectin (EDA-FN, green) and collagen I (red) in PCLS treated with fibrotic cocktail was reduced after addition of DHEA (FC + D150). Nucleus staining with DAPI (blue). Pictures were taken at 10X magnification. (–) scale represent 500 µm.


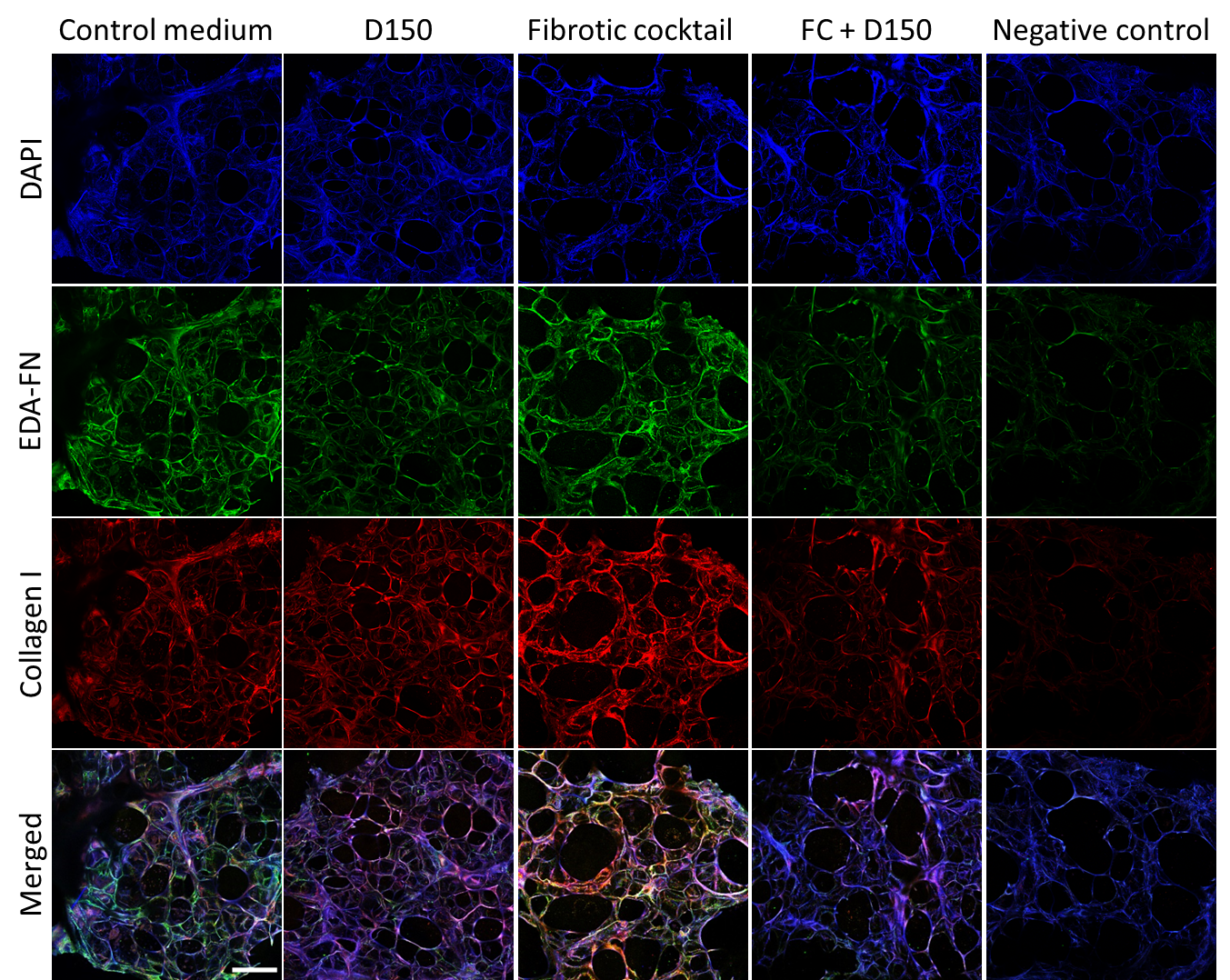


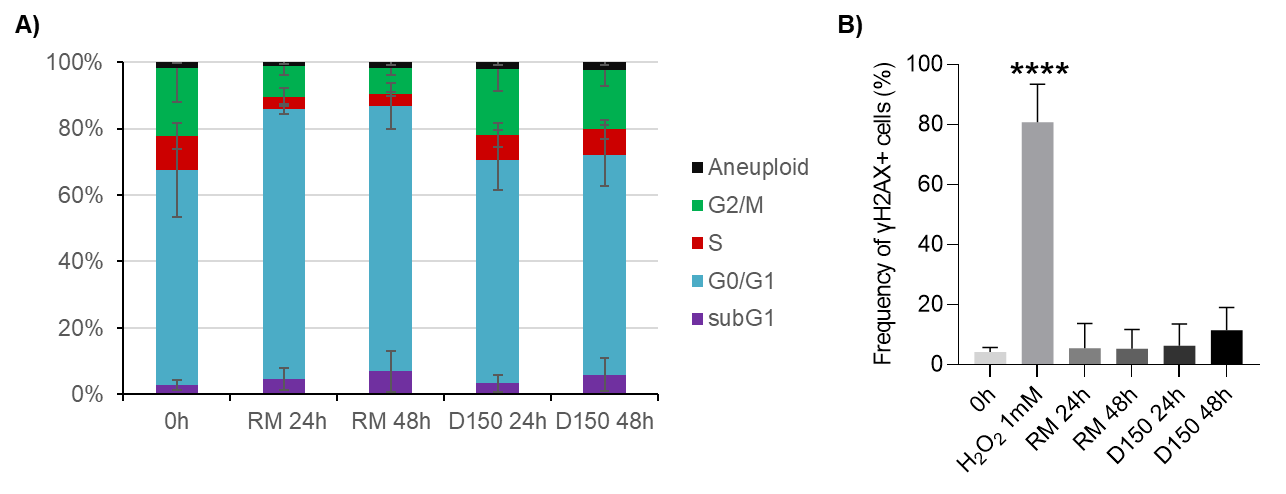
**Figure S4.** **Effect of DHEA on the cell cycle and DNA damage.** A) Cell cycle distribution of control lung fibroblasts (n=3) at the beginning of the experiment (0h) and after treatment with DHEA (D150) or in resting medium (RM) for 24 and 48h. B) Frequency of γH2AX+ cells in lung fibroblasts treated with/without DHEA. (****) p<0.0001. compared to the positive control of DNA damage (H_2_O_2_ 1mM).
